# Supplementary material for: Mycosporine-Like Amino Acids (MAAs) in Time-Series of Lichen Specimens from Natural History Collections
Source: Molecules. 2019 Mar 19;24(6):1070. doi: 10.3390/molecules24061070 (PMC6471344; doi:10.3390/molecules24061070)
Supplement: Supplementary file 1 [file molecules-24-01070-s001.pdf]

molecules

Mycosporine-like amino acids (MAAs) in time series of lichen specimens from Natural History Collections

Marylène Chollet-Krugler[1], Thi Thu Tram Nguyen[1,2], Aurelie Sauvager[1], Holger Thüs[3,4*] and Joël Boustie[1,*]

1 Univ Rennes, CNRS, ISCR (Institut des Sciences Chimiques de Rennes) - UMR 6226, F-35000 Rennes, France ; joel.boustie@univ-rennes1.fr
2 Department of Chemistry, Faculty of Science, Can Tho University of Medicine and Pharmacy, Vietnam ; ntttram@ctump.edu.vn
3 State Museum of Natural History Stuttgart; holger.thues@smns-bw.de
4 The Natural History Museum London
* Correspondence: joel.boustie@univ-rennes1.fr; holger.thues@smns-bw.de

Supplementary Materials

Table 1. Collection details and analysis results for the lichens specimens in this study (mean values ± SD, mg/g of dw. extract, n = 2). For specimens where only the accession time into a larger public herbarium instead of the collecting time is known, the date is marked by an asterisk (*).

Collection Number	Collector	Origin	Year	Mycosporine glutaminol [mg/g ± STW]	Mycosporine glutamicol [mg/g ± STW]
BM001085252	W.E. Ahles	Germany, Odenwald	1861	n.d.	15.73 (± 3.39)
BM001085371	W.E. Ahles	Germany, Odenwald	1861	n.d.	14.22 (± 0.16)
BM001085263	G. Davies	UK, Devon	1864	n.d.	27.04 (± 2.26)
BM001085373	H.B. Holl	UK, Devon	1886*	n.d.	10.65 (± 2.26)
BM001085239	H.B. Holl	UK, Devon	1886*	n.d.	10.07 (± 0.53)
BM000974964	H.B. Holl	UK, Devon	1886*	n.d.	10.52 (± 1.34)
BM001085262	H.B. Holl	UK, Devon	1886*	n.d.	16.57 (± 4.44)
BM001085261	H.B. Holl	UK, Devon	1886*	n.d.	27.78 (± 1.91)
BM000974963	H.B. Holl	UK, Devon	1886*	n.d.	19.51 (± 2.68)
BM000974962	H.B. Holl	UK, Wales	1886*	n.d.	13.09 (± 1.22)
BM000974961	W. Johnson	UK, Cumberland	1894	n.d.	13.22 (± 2.77)
BM001085361	J. Hartz, C. Ostenfeld	Faroer Islands	1897	n.d.	4.56 (± 0.84) under LOQ
BM001085235	F.W. Zopf	Germany, Harz	1897	n.d.	13.60 (± 1.43)

BM001085376	C. Lelt	Ireland	1905	n.d.	14.08 (± 0.58)
BM001085243	J.A. Crabbe	UK, Outer Hebrides	1905	n.d.	n.d.
BM001085243	J.A. Crabbe	UK, Outer Hebrides	1905	n.d.	n.d.
BM001085368	E. Parfitt	UK, (Devon) Dartmoor	1905	n.d.	n.d.
BM000974960	H.H. Knight	UK, Carmarthenshire	1908	n.d.	16.89 (± 1.38)
BM001085236	A. Willi	Austria, Tirol	1920	n.d.	18.06 (± 3.03)
BM001085375	A. Willi	Austria, Tirol	1920	n.d.	8.5 (± 0.1) under LOQ
REN-ABB_B002_C08_01	H. Des Abbayes	France, Côtes D'Amor	1927	n.d.	9.07 (± 0.86)
REN-ABB_B002_C08_02	H. Des Abbayes	France, Côtes d'Armor	1931	n.d.	8.8 (± 0.62) under LOQ
REN-ABB_B002_C08_03	H. Des Abbayes	France, Vendée	1931	n.d.	21.46 (± 1.39)
REN-ABB_B002_C08_08	H. Des Abbayes	France, Pyrénées Orientales	1933	n.d.	9.95 (± 4.21)
REN-ABB_B002_C08_04	H. Des Abbayes	France, Finistère	1933	n.d.	14.48 (± 3.66)
REN-ABB_B002_C08_05	H. Des Abbayes	France, Côtes d'Armor	1933	n.d.	26.4 (± 5.58)
REN-ABB_B002_C08_06	H. Des Abbayes	France, Loire-Atlantique	1933	n.d.	14.27 (± 1.98)
BM001085256	V. Räsänen	Finland, Karelia	1936	n.d.	17.77 (± 1.73)
BM001085255	Fagerström	Finland, Karelia	1947	n.d.	12.92 (± 1.20)
BM001085244	P.W. James	UK, Pembrokeshire	1958	n.d.	8.53 (± 0.91)
BM001085242	P.W. James	UK, Pembrokeshire	1958	n.d.	9.24 (± 1.06)
BM001085245	P.W. James	UK, West Sutherland	1959	n.d.	7.14 (± 0.82) under LOQ
BM001085245	P.W. James	UK, West Sutherland	1959	n.d.	n.d.
BM001085241	P.J. Hunt	UK, Outer Hebrides	1961	n.d.	15.07 (± 1.20)
BM001085237	H. Ullrich	Germany, Harz	1963	n.d.	22.64 (± 0.98)
REN_MAS_73_01	L.J.-C. Massé	France, Côtes d'Armor	1963	n.d.	14.93 (± 1.58)
BM001085248	T.D.V. Swinscow	UK, Devon	1963	n.d.	12.77 (± 0.12)

BM001085249	P.W. James	UK, Carmarthenshire	1965	n.d.	10.96 (± 3.00)
BM001085246	F.J. Walker	UK, Cumberland	1979	n.d.	6.75 (± 0.82) under LOQ
JB/001/08/2005	J.L. & B.Martin	France, Gare	2005	n.d.	20.07 (± 1.01)
REN-HL L10/11-5	M.Millot	France, Corrèze	2005	11.28 (± 4.72)	4.72 (± 2.64) under LOQ
JB/010/07/2005	J. La Gabrielle	France, HautRhin	2005	3.82 (± 0.76)	22.08 (± 1.07)
JB/001/08/2006	J.L. & B.Martin	France, Haut-Rhin	2006	n.d.	27.1 (± 0.52)
BM000731396	H. Thüs	Germany, Odenwald	2006	2.8 (± 0.19)	21.00 (± 0.61)
BM000920143	V. Howden	UK, Wales	2006	3.65 (± 1.32)	9.36 (± 1.79)
BM001085250	C. Gueidan	UK, Devon	2010	15.54 (± 0.98)	16.83 (± 0.44)
JB/001/07/2012	M. Bertrand	France, Pyrenées Orientales	2012	14.68 (± 0.25)	11.74 (± 0.61)
REN-JB/001/09/2013	J.Y. Monnat	France, Brittany	2013	13.31 (± 1.32)	9.28 (± 1.21)
